# Demographic, Clinical, Psychosocial, and Behavioral Predictors of Continuous Glucose Monitor Use in Adults with Type 2 Diabetes

**DOI:** 10.1007/s11606-024-09101-1

**Published:** 2024-10-25

**Authors:** Emily L. Morrow, Andrew J. Spieker, Robert A. Greevy, McKenzie K. Roddy, Lindsay S. Mayberry

**Affiliations:** 1 Division of General Internal Medicine & Public Health, Department of Medicine, Vanderbilt University Medical Center, Nashville, USA; 2 Center for Health Behavior & Health Education, Vanderbilt University Medical Center, Nashville, TN USA; 3 Department of Biostatistics, Vanderbilt University Medical Center, Nashville, TN USA

**Keywords:** continuous glucose monitoring, type 2 diabetes, behavioral characteristics, psychosocial characteristics, HbA1c

## Abstract

**Background:**

Continuous glucose monitor (CGM) use is increasing rapidly among people with type 2 diabetes, although little is known about predictors of CGM use beyond clinical and demographic information available in electronic medical records. Behavioral and psychosocial characteristics may also predict CGM use.

**Objective:**

We examined clinical, psychosocial, and behavioral characteristics that may predict CGM use in adults with type 2 diabetes.

**Design:**

This longitudinal observational study comprised a secondary analysis of data collected in a larger trial. Enrollment included HbA1c tests and surveys assessing demographic, clinical, psychosocial, and behavioral characteristics. We queried participants regarding their CGM use during the study on their final self-report surveys, 15 months post-enrollment.

**Participants:**

Participants were 245 community-dwelling adults with type 2 diabetes recruited from primary care.

**Approach:**

We used logistic regression to predict CGM use during the 15-month trial period from baseline characteristics.

**Key Results:**

Around one-third of participants (37.1%; 91/245) started CGM. Predictors of starting CGM in bivariate models included younger age, higher socioeconomic status, insulin use, higher HbA1c, and more diabetes distress. When including all potential predictors in a single multivariable model, only younger age (aOR = 0.95, *p* = 0.001), insulin use (aOR = 2.33, *p* = 0.006), and higher socioeconomic status (aOR = 0.44, *p* = 0.037) were significant predictors. Despite the association between higher HbA1c and CGM use, neither diabetes self-care behaviors nor diabetes self-efficacy significantly predicted CGM use. Of participants who tried a CGM, 14.3% (13/91) had stopped, with cost being the most-cited reason.

**Conclusions:**

Even when including behavioral and psychological characteristics, younger age, using insulin, and higher socioeconomic status remain key predictors of CGM use. These findings emphasize the importance of access and affordability for people who may benefit from CGM. Providers should not bias their introduction of CGM towards those with (perceived or actual) optimal or sub-optimal self-care behaviors.

Despite improvements in diabetes medications, fewer than half of people with type 2 diabetes meet glycemic targets over time.^1^ Virtually monitoring glucose via continuous glucose monitors (CGM) may promote behavior change, improve glycemic management, and inform insulin dosing.^2–4^ CGM is an established standard of care in type 1 diabetes.^5^ Recent studies have shown that CGM use may also support people with type 2 diabetes in lowering hemoglobin A1c (HbA1c), maintaining greater time in range, and improving quality of life.^2,4–11^

Improved accessibility, technology, insurance coverage, and support have increased CGM uptake in people with type 2 diabetes.^4,12^ Mayberry et al.^12^ showed a rapid increase between 2019 and 2021 at Vanderbilt University Medical Center (VUMC), a large academic medical center in the mid-South US. From 2020 to 2021, the growth rate of CGM users among outpatients with type 2 diabetes was 36% overall, with the largest growth (125%) in primary care.^19^

Early work has shown that people with type 2 diabetes who use CGM are younger, have higher HbA1c, are more likely to use insulin, and are more likely to receive endocrinology care than non-users.^12^ In type 1 diabetes, age ≥ 25 years,^13^ white race,^14^ higher socioeconomic status,^14^ and private health insurance^14^ are associated with CGM use, along with more diabetes self-care behaviors,^14^ less diabetes distress,^15^ and fewer depressive symptoms.^14^ However, studies assessing *predictors* measured before use, rather than *correlates* or *outcomes* measured alongside use, are limited. In type 1 diabetes, we are aware of one study^16^ investigating characteristics associated with interest prior to using CGM, which included having lower HbA1c, using an insulin pump, more diabetes self-care behaviors, less diabetes-related family conflict, and higher quality of life.^16^

To date, no studies have examined behavioral and psychological characteristics that predict CGM use in type 2 diabetes. We had a unique opportunity to conduct a secondary analysis of data from a larger, 15-month clinical trial with participants with type 2 diabetes recruited from primary care at VUMC during the identified period of increased CGM uptake. In this longitudinal observational study, we examined clinical, psychosocial, and behavioral characteristics that may predict CGM use.

## METHODS

This is a secondary analysis of data collected from adults with type 2 diabetes in a larger randomized controlled trial evaluating a mobile phone–delivered diabetes self-management support intervention (Family/friend Activation to Motivate Self-care, FAMS).^17^ The study protocol was approved by the Vanderbilt Institutional Review Board. We recruited outpatients receiving primary care from VUMC with a most recent HbA1c value of ≥ 7.5%. All participants received informational print materials and results from HbA1c tests throughout the study (i.e., enhanced care as usual as control condition). Participants in the intervention group also received monthly coaching calls and daily text message support to address chosen self-care goals (i.e., diet, exercise, stress, management). The intervention did not address CGM because CGM use was not common among adults with type 2 diabetes during its development. We had no reason to believe being assigned to the intervention group would be related to CGM use; however, all participants—regardless of assigned condition—joined a trial about self-management support and therefore may be interested in other supports, such as CGM. Outcomes from the larger trial have been published.^18,19^ .

The timeline of the larger trial aligned with the rapid increase in CGM uptake in adults with type 2 diabetes at VUMC, per prior observational research using electronic medical record data.^12^ Baseline trial visits occurred May 2020–October 2021, and the 15-month assessments during which participants reported on their CGM use in the prior 12 months occurred October 2021–March 2022; therefore, trial participants reported on their use of CGM from the end of 2020 through early 2022. In the Mayberry et al.^12^ study of 30,585 adults with type 2 diabetes receiving outpatient diabetes care at VUMC, only 1.5% used CGM prior to January 2021. For each month in 2021, a mean of 90.5 (SD 12.5) people with type 2 diabetes started using CGM, with a 125% growth rate in primary care prescriptions relative to 2020.^12^ The sample for this study was recruited from primary care at VUMC, so based on published trends, CGM use in this trial sample prior to the study period was likely rare.

### Participant Eligibility

Participants were recruited from primary care clinics at VUMC and were diagnosed with type 2 diabetes, between the ages of 18 and 75 years old, and English speakers. Participants’ most recent HbA1c value was ≥ 7.5% within the prior 183 days. Participants were community dwelling (e.g., not in a nursing facility), prescribed at least one daily diabetes medication, and owned a mobile phone. Participants were excluded for concurrent hospice or dialysis services, congestive heart failure, concurrent cancer treatment, pregnancy, dementia, or schizophrenia; or for self-disclosed recent or ongoing emotional, physical, or sexual abuse. These exclusionary characteristics may reduce focus on diabetes management or impede participants’ ability to respond to self-report measures.

### Data Collection Schedule

Participants completed self-report surveys and an HbA1c test at enrollment. Given the period of increased CGM uptake that coincided with the trial, we queried participants regarding their CGM use during the study period on their final self-report surveys, 15 months post-enrollment. This secondary analysis is restricted to 74% (245/329) of participants who answered questions about CGM use during the trial at the 15-month follow-up.

### Measures

#### Characteristics at Enrollment

##### Demographic and Clinical

We assessed age, gender, years of education, race, ethnicity, diabetes duration, household income, insurance status, and insulin use via self-report. We confirmed insulin use with the electronic medical record. HbA1c was collected either via venipuncture or point-of-care device during regular medical care or via CoreMedica laboratory mail-in test kits (validated against venipuncture).^20^

##### Psychological

We assessed the following psychosocial characteristics using validated self-report measures: diabetes distress (Problem Areas in Diabetes [PAID]^21^ with scores ranging from 0 to 100, higher scores indicate more diabetes distress, scores ≥ 40 indicate clinically significant distress), depressive symptoms (Patient Health Questionnaire [PHQ-8]^22^ with scores ranging from 0 to 24, higher scores indicate more depressive symptoms, scores ≥ 10 indicate clinically significant symptoms), and diabetes self-efficacy (Perceived Diabetes Self-Management Scale [PDSMS]^23^ with scores ranging 8 to 40, higher scores indicate more self-efficacy).

##### Behavioral

We assessed the following behavioral characteristics using validated self-report measures: medication adherence (Adherence to Refills and Medications Scale for Diabetes [ARMS-D]^24^ with scores ranging 11–44, reverse-coded such that higher scores indicate more adherence), dietary behavior (Use of Information for Dietary Decision Making scale from the Personal Diabetes Questionnaire [PDQ]^25^ with scores ranging 1–6, higher scores indicate more use of dietary information in decision making), and physical activity (modified version of the Rapid Assessment of Physical Activity [RAPA]^26^ with scores ranging from 0 to 6533 metabolic equivalent of task minutes per week, higher scores indicate more physical activity).

#### CGM Use

The 15-month follow-up surveys asked participants whether they currently use a CGM (yes/no). For participants who were not currently using a CGM, we asked whether they had used a CGM in the prior 12 months (yes/no). CGM use was operationalized as using a CGM during the last 12 months of the 15-month trial, regardless of having stopped or continued use. We asked participants who had stopped using a CGM for their reasons. Respondents could select multiple provided reasons and/or select “other” and type their reason.

### Data Analysis

We used logistic regression to test associations between characteristics at enrollment and CGM use during the study period. We tested each predictor in bivariate models (i.e., including CGM use [yes/no] as the outcome in separate models for each potential predictor). Then, we tested for conditionally independent effects in a model including all potential predictors of CGM use. We collapsed some categories and variables (see footnotes on Table 2) for the multivariable model to preserve degrees of freedom. To address missing data, we used multiple imputation by chained equations with *M* = 200 iterations for the multivariable model. Finally, we identified demographic and clinical characteristics of participants who had stopped using CGM and descriptive information on their reported reasons for stopping.

#### Sensitivity Analysis for Intervention Condition

The self-management support intervention evaluated in the larger RCT did not target or encourage CGM use,^17^ because CGM use was uncommon in people with type 2 diabetes when the study started. Nonetheless, we conducted a sensitivity analysis to determine if condition assignment was associated with CGM use during the study. Of the 91 CGM users in the RCT, 57.1% (*n* = 52/91) were assigned to the intervention group, and 42.9% (*n* = 39/91) were assigned to the control group. First, we used bivariate logistic regression to test whether condition (intervention/control) predicted CGM use during the study period (yes/no). Second, we ran the multivariable model with intervention condition included as a predictor. Condition was not associated with CGM use in the bivariate (*p* = 0.14) nor multivariable (aOR = 1.48, *p* = 0.19) models, and direction and significance of other predictors were unchanged. Therefore, we report results from our a priori analysis, which did not include condition and preserved degrees of freedom.

## RESULTS

Participants were on average 57.1 ± 10.8 years old, 38.0% reported minoritized race or ethnicity, and 50.2% were male. Average diabetes duration was 11.5 ± 8.1 years, 38.8% used insulin, and average HbA1c was 8.6% ± 1.7. Table 1 shows characteristics by CGM use.

### Predictors of CGM Use

Over a third (37.1%) of participants (91/245) reported using a CGM during their study experience. In bivariate models, younger people (*p* < 0.001), people with higher HbA1c at enrollment (*p* = 0.003), people with higher household income (*p* = 0.031), and people who used insulin at enrollment (*p* = 0.004) were more likely to use CGM during the study period. People who reported more diabetes distress at enrollment (*p* = 0.043) were also more likely to use CGM during the study period.

Separately, we examined diabetes distress and depressive symptoms categorically using established cut points. People with clinically elevated diabetes distress at enrollment (*n* = 112/245) were non-significantly more likely to use a CGM during the study period (*p* = 0.070). Having elevated depressive symptoms at enrollment (*n* = 47/245) was not associated with CGM use (*p* = 0.870).

**Table 1:** Characteristics of Study Participants by CGM Use, and Unadjusted Tests of Difference

	*n*	CGM users	Non-users	*p* value
		(*n* = 91)	(*n* = 154)	
		Mean (SD) or *n* (%)		
**Demographic characteristics**				
Age (years)	245	53.9 (10.4)	59.0 (10.7)	< 0.001*
Gender	245			
Male (*referent*)		45 (49.5%)	78 (50.7%)	
Female		45 (49.5%)	75 (48.7%)	0.883
Other		1 (1.1%)	1 (0.7%)	0.700
Education (years)	241	15.4 (3.7)	15.4 (2.5)	0.959
Race and ethnicity	245			
Non-Hispanic White (*referent*)		51 (56.0%)	101 (65.6%)	
Non-Hispanic Black		29 (31.9%)	32 (20.8%)	0.058
Hispanic		7 (7.7%)	12 (7.8%)	0.775
Other		4 (4.4%)	9 (5.9%)	0.838
Household income	234			
< $35,000 annually (*referent*)		10 (11.5%)	34 (23.1%)	
$35,000 or more annually		77 (88.5%)	113 (76.9%)	0.031*
Insurance status	239			
No private insurance (*referent*)		12 (13.5%)	36 (24.0%)	
Private insurance		77 (86.5%)	114 (76.0%)	0.053
**Clinical characteristics**				
Diabetes duration (years)	242	12.0 (8.2)	11.1 (8.0)	0.397
HbA1c (%)	230	9.0 (1.9)	8.3 (1.4)	0.003*
Insulin status	243			
Oral meds only (*referent*)		42 (46.2%)	106 (69.7%)	
Oral and insulin		39 (42.9%)	43 (28.3%)	0*.*004*
Insulin only		10 (10.9%)	3 (2.0%)	0.002*
**Psychological characteristics**				
Diabetes distress	242	41.6 (26.3)	34.6 (25.5)	0.043*
Depressive symptoms	240	5.6 (5.0)	5.6 (5.4)	0.970
Diabetes self-efficacy	242	25.4 (7.0)	25.5 (6.7)	0.939
**Behavioral characteristics**				
Medication adherence	242	39.8 (3.6)	40.6 (3.6)	0.095
Dietary behavior	242	2.9 (1.4)	3.1 (1.5)	0.192
Physical activity	242	740.2 (831.1)	720.6 (905.2)	0.866

Unadjusted bivariate logistic regression models used for tests of difference

*SD*, standard deviation

^*^Significant at *p* < 0.05

In the multivariable model testing for conditionally independent effects (Table 2), only younger age (aOR = 0.95, *p* = 0.001), insulin use (aOR = 2.33, *p* = 0.006), and higher socioeconomic status (aOR = 0.44, *p* = 0.037) were significant predictors of CGM use.

**Table 2:** Adjusted Odds Ratios from the Multivariable Model Predicting CGM Use

	Adjusted odds ratio	
	[95% CI]	*p* value
**Demographic characteristics**		
Age (years)	0.96 [0.92, 0.99]	0.008*
Gender		
Male (*referent*)		
Non-male	0.97 [0.52, 1.79]	0.917
Education (years)	1.00 [0.90, 1.11]	0.995
Race and ethnicity^a^		
Non-Hispanic White (*referent*)		
Non-Hispanic Black	0.93 [0.43, 2.00]	0.849
Other	0.65 [0.26, 1.62]	0.353
Low socioeconomic status^b^	0.44 [0.21, 0.95]	0.037*
**Clinical characteristics**		
Diabetes duration (years)	1.03 [0.99, 1.08]	0.125
HbA1c (%)	1.17 [0.97, 1.41]	0.110
Insulin status	2.46 [1.34. 4.53]	0.004*
Not using insulin (*referent*)		
Using insulin	2.46 [1.34, 4.53]	0.004*
**Psychological characteristics**		
Diabetes distress	1.01 [0.99, 1.02]	0.351
Depressive symptoms	0.98 [0.91, 1.05]	0.485
Diabetes self-efficacy	1.01 [0.97, 1.07]	0.466
**Behavioral characteristics**		
Medication adherence	0.98 [0.90, 1.07]	0.696
Dietary behavior	0.85 [0.69, 1.05]	0.124
Physical activity	1.00 [1.00, 1.00]	0.575

Multivariable model used imputed data, *n* = 245

*CI*, confidence interval

^a^In the multivariable model, race and ethnicity were coded as non-Hispanic White, non-Hispanic Black, or other, which included Hispanic

^b^In the multivariable model, income and insurance status were combined into a single proxy socioeconomic status variable (low/high), such that people with incomes lower than $35,000 annually or who did not have private insurance were coded as likely having low socioeconomic status

^*^Significant at *p* < 0.05

### Barriers to CGM Use

Of participants who used CGM during the study period, 14.3% (13/91) reported they had stopped. About a quarter (24.1%; 7/29) of non-Hispanic Black CGM users stopped, as compared to 11.8% (6/51) of non-Hispanic White CGM users and 0.0% (0/13) of Hispanic and other race(s) users. A fifth (21.4%; 9/42) of CGM users taking oral medications only stopped, as compared to only 8.2% (4/49) of those taking insulin. The most common reason for stopping was cost (*n* = 9, 69.2%), followed by problems with adhesive (*n* = 4, 30.8%), pain or discomfort (*n* = 2, 15.4%), problems with connectivity (*n* = 2, 15.4%), needing to change the sensor too often (*n* = 2, 15.4%), and dislike of having a CGM on the body (*n* = 2, 15.4%). One participant (7.7%) felt the data were overwhelming, and one participant (7.7%) reported that the CGM was not accurate, requiring verification with a glucometer.

## DISCUSSION

People with type 2 diabetes may benefit from CGM use for regular feedback and a personalized approach to glycemic management,^27^ so we sought to illuminate who is starting CGM use, who is stopping CGM use, and their reasons. We had a unique opportunity to assess numerous potential predictors of CGM use in a sample of adults with type 2 diabetes receiving primary care, representing a category of patients within our medical system who have seen the most growth in CGM use and who most need diabetes management support (i.e., recruited with elevated HbA1c, interested in self-management support). We found that 37.1% had used a CGM, and 14.3% of those had stopped using it. Cost was the most-cited reason for stopping CGM use. This was notable given CGM users had higher socioeconomic status than non-users. Socioeconomic predictors of CGM use have also been important in type 1 diabetes.^14^ This association is likely attributable to the rapidly changing, and at times challenging, landscape of accessing CGM, including health insurance policies and changes thereof and the sometimes-burdensome requirements of obtaining new devices and navigating device failures.

CGM use was predicted by younger age, insulin use, higher HbA1c, and higher diabetes distress, suggesting people with type 2 diabetes who try CGM may want or need more support managing diabetes. This was consistent with the emerging literature on factors associated with CGM use in people with type 1 and type 2 diabetes.^12,14^ Specifically, insulin dosing may require more frequent blood glucose testing, which may be less burdensome with CGM.^12^ Some insurers also require that people with type 2 diabetes be on insulin therapy to have CGM covered by insurance, which may be related to the high prevalence of insulin therapy in CGM users. CGM may increasingly be offered to patients needing additional support due to increased awareness among patients and primary care providers of the benefits of CGM use for improving HbA1c and supporting diabetes management. Younger age may stand alone as a driving factor due to the technological demands of CGM.^28,29^

Contrary to our expectations, neither diabetes self-care behaviors nor diabetes self-efficacy significantly predicted CGM use, despite the difference in HbA1c among users and non-users. Providers should consider CGM as a potential tool for anyone with type 2 diabetes and not bias their introduction of CGM towards those with (perceived or actual) optimal or sub-optimal self-care behaviors. Consistent with prior work,^12^ we did not find evidence of CGM use disparities by gender, race, nor ethnicity. However, about a quarter of non-Hispanic Black participants who began using CGM in this sample stopped, which was a larger proportion than in other racial/ethnic groups. Future research should consider methods to not only increase initial access to CGM, but also to reduce disparities in use and outcomes over time.

### Potential Limitations

Some characteristics of the trial sample (e.g., the requirement to own a cell phone, English speakers) may have differed from the general population of people with type 2 diabetes. Conversely, this sample was diverse regarding race, ethnicity, and socioeconomic status, which allowed us to evaluate these characteristics as predictors. We relied on retrospective, self-reported CGM use, so there may have been some misspecification of CGM use at baseline, but use of CGM at the time of study enrollment was rare across the health system, suggesting misspecification error was likely insufficient to bias findings. In this sample, we did not have information about patterns of CGM use (maintenance over time, frequency of use, reading and applying data), and our analyses combined people who had stopped using CGM with those who continued use. Future work should consider how behavioral and psychological predictors relate to CGM use and outcomes over time.^30^

## CONCLUSIONS

This study is the first to examine behavioral and psychological predictors of CGM use in type 2 diabetes. Even when including these variables, age, insulin use, and socioeconomic status remain key predictors. Higher HbA1c and more diabetes distress also predict using a CGM. More work is needed to understand how individual behavioral and psychological profiles (e.g., self-efficacy and self-management) predict CGM use and outcomes, as these variables may work together multidimensionally to influence use of CGM.

## Data Availability

Available from the corresponding author upon reasonable request.
